# Activity of AMP2041 against human and animal multidrug resistant *Pseudomonas aeruginosa* clinical isolates

**DOI:** 10.1186/s12941-017-0193-1

**Published:** 2017-03-23

**Authors:** Clotilde Silvia Cabassi, Andrea Sala, Davide Santospirito, Giovanni Loris Alborali, Edoardo Carretto, Giovanni Ghibaudo, Simone Taddei

**Affiliations:** 1 Department of Veterinary Science, University of Parma, Via del Taglio 10, 43126 Parma, Italy; 2 Istituto Zooprofilattico Sperimentale della Lombardia e dell’Emilia Romagna, Via Bianchi 7/9, 25124 Brescia, Italy; 3 Arcispedale S. Maria Nuova, Viale Risorgimento 80, 42123 Reggio Emilia, Italy; 4 Clinica Veterinaria Malpensa, Via Marconi 27, 21017 Samarate, VA Italy

**Keywords:** Antimicrobial peptide, Pseudomonas aeruginosa, Cinical isolates, Multidrug resistance, Bacterial membrane damage

## Abstract

**Background:**

Antimicrobial resistance is a growing threat to public health. *Pseudomonas aeruginosa* is a relevant pathogen causing human and animal infections, frequently displaying high levels of resistance to commonly used antimicrobials. The increasing difficulty to develop new effective antibiotics have discouraged investment in this area and only a few new antibiotics are currently under development. An approach to overcome antibiotic resistance could be based on antimicrobial peptides since they offer advantages over currently used microbicides.

**Methods:**

The antimicrobial activity of the synthetic peptide AMP2041 was evaluated against 49 *P. aeruginosa* clinical strains with high levels of antimicrobial resistance, isolated from humans (n = 19) and animals (n = 30). In vitro activity was evaluated by a microdilution assay for lethal dose 90% (LD_90_), while the activity over time was performed by time-kill assay with 12.5 µg/ml of AMP2014. Evidences for a direct membrane damage were investigated on *P. aeruginosa* ATCC 27853 reference strain, on animal isolate PA-VET 38 and on human isolate PA-H 24 by propidium iodide and on *P. aeruginosa* ATCC 27853 by scanning electron microscopy.

**Results:**

AMP2041 showed a dose-dependent activity, with a mean (SEM) LD_90_ of 1.69 and 3.3 µg/ml for animal and human strains, respectively. AMP2041 showed microbicidal activity on *P. aeruginosa* isolates from a patient with cystic fibrosis (CF) and resistance increased from first infection isolate (LD_90_ = 0.3 μg/ml) to the mucoid phenotype (LD_90_ = 10.4 μg/ml). The time-kill assay showed a time-dependent bactericidal effect of AMP2041 and LD_90_ was reached within 20 min for all the strains. The stain-dead assay showed an increasing of membrane permeabilization and SEM analysis revealed holes, dents and bursts throughout bacterial cell wall after 30 min of incubation with AMP2041.

**Conclusions:**

The obtained results assessed for the first time the good antimicrobial activity of AMP2041 on *P. aeruginosa* strains of human origin, including those deriving from a CF patient. We confirmed the excellent antimicrobial activity of AMP2041 on *P. aeruginosa* strains derived from dog otitis. We also assessed that AMP2041 antimicrobial activity is linked to changes of the *P. aeruginosa* cell wall morphology and to the increasing of membrane permeability.

## Background

*Pseudomonas aeruginosa* is a relevant pathogen causing human and animal infections. In humans, severe *P. aeruginosa* infections usually occur in immunocompromised patients and in nosocomial setting. *P. aeruginosa* infection often follow surgery or invasive procedures and causes mainly pneumonia and septicaemia. *P. aeruginosa* may also cause mild illnesses in healthy people, in which skin, ear and eye infections can occur. Moreover, *P. aeruginosa* is the major pathogen in the cystic fibrosis (CF). In CF, chronic *P. aeruginosa* infections occur in up to 85% of CF patients and the *P. aeruginosa* strains involved develop antibiotic resistance and phenotypic changes, from first infection to chronic infection and mucoid phenotype. These phenotypical changes could play a major role in the persistence of *P. aeruginosa* infections in CF patients [1]. Antibiotic resistance and the persistence of the organisms despite therapy once chronic infection has been established, is leading to the search for more effective therapeutic approaches [1].

*Pseudomonas aeruginosa* also cause diseases in both livestock and companion animals, including otitis and urinary tract infections in dogs, mastitis in dairy cows and endometritis in horses [2]. Resistance phenotypes are more frequent in dogs and multi-drug resistant (MDR) *P. aeruginosa* seem to emerge mainly in those suffering from otitis. Antimicrobial resistance in animal *P. aeruginosa* infections should be closely monitored in the future, in line with possible animal-to-human transfers between pets and owners [2]. *P. aeruginosa* is naturally resistant to many classes of drugs and its capacity to rapidly develop resistance during treatment is a frequent source of therapeutic failures. *P. aeruginosa* is one of the six ESKAPE pathogens, reported by the Infectious Diseases Society of America, that urgently require novel therapies [3]. Rates of antibiotic resistance in *P. aeruginosa* are increasing worldwide even if the true frequency of infections caused by MDR *P. aeruginosa* is difficult to estimate. A review of studies reporting on MDR, extensively-drug resistant (XDR) and pan-drug resistant (PDR) *P. aeruginosa* infections revealed that aminoglycosides, antipseudomonal penicillins, cephalosporins, carbapenems and fluoroquinolones [4] have become ineffective as first line agents. The multidrug resistance of *P. aeruginosa* could be mediated by several mechanisms including multidrug efflux systems, enzyme production, outer membrane protein loss and target mutations [5]. The spread of antimicrobial resistance increase human and animal health hazard worldwide, thus makes mandatory the investigation of novel approaches to cover the therapeutic shortfall. In this view, one of the actions put forward in the European Commission Action Plan is to develop effective antimicrobials or alternatives for treatment of human and animal infections and to reinforce research to develop innovative means to combat antimicrobial resistance [6]. Antimicrobial peptides offer potential advantages over currently used classes of drugs. They may counteract pathogenic challenge by rapid, broad spectrum, microbicidal activity [7], targeting multiple pathogens with one treatment. Moreover, antimicrobial peptides may have the potential to ultimately reduce the rate of emergence of resistant microorganisms, since selective pressure is not focused to a single specific molecular target. Further, antimicrobial peptides could also be potentially used in conjunction with conventional antibiotics as part of a “combination therapy” to create an additive or synergistic effect.

The antimicrobial peptide AMP2041 is a cyclic antimicrobial peptide, belonging to a novel family of antimicrobial cationic peptides, which showed good antimicrobial activity against a panel of different Gram-positive and Gram-negative bacterial pathogens of animal origin [8]. The activity of AMP2041 against *P. aeruginosa* ATCC 27853 was also demonstrated, as well as additivity in combination with levofloxacin [9]. We hypothesized that the reported antimicrobial activity derived from a bacterial membrane damage, but a direct membrane damage was not previously investigated for *P. aeruginosa*.

The aim of the present work was to evaluate the antimicrobial activity of AMP2041 on different MDR, PDR and XDR *P. aeruginosa* clinical isolates of human origin, including five different phenotypes of *P. aeruginosa* derived from a single patient with cystic fibrosis, and on clinical MDR *P. aeruginosa* clinical isolates deriving from animals, mainly dogs with otitis. Further, we investigated the evidence for a direct membrane damage on *P. aeruginosa* ATCC 27853 reference strain.

## Methods

### Bacterial strains and antibiotic susceptibility

Isolates and their biochemical profiles, obtained by API System (bioMérieux, Marcy l’Etoile, France), are reported in Tables 1, 2 and 3. Antibiotic susceptibility tests were performed using the system Vitek2 (bioMérieux, Marcy l’Etoile, France) and/or the Kirby–Bauer method (antibiotic disks provided by Mast Diagnostics Germany, Oxoid, UK). *P. aeruginosa* drug-resistant strains were defined following the European Centre for Disease Prevention and Control (ECDC) guidelines [10]. The following classes of antimicrobials were tested: aminoglycosides, carbapenems, cephalosporins, fluoroquinolones, penicillins, monobactams, phosphonic acids, polymyxins. The resistance profiles of the isolates are reported in Tables 1, 2 and 3.

**Table 1:** *Pseudomonas aeruginosa* human clinical isolates

Reference number	ID number	Source	API20E	Sample	Resistance profile
[1]	PA-H 1	Human	2216004	Urine	XDR
[2]	PA-H 10	Human	2217046	Blood	MDR
[3]	PA-H 24	Human	2216046	Blood	XDR
[4]	PA-H 25	Human	2206046	Urine	MDR
[5]	PA-H 37	Human	2206046	Blood	MDR
[6]	PA-H 45	Human	2210004	Urine	MDR
[7]	PA-H 47	Human	2217046	Blood	MDR
[8]	PA-H 52	Human	2206006	Blood	MDR
[9]	PA-H 56	Human	2206046	Blood	XDR
[10]	PA-H 58	Human	2206006	Blood	MDR
[11]	PA-H 71	Human	2206046	Blood	MDR
[12]	PA-H 37/2	Human	2206006	Blood	MDR
[13]	PA-H 45/2	Human	2206006	Urine	MDR
[14]	PA-H 14	Human	2206046	Blood	MDR

**Table 2:** *Pseudomonas aeruginosa* clinical isolates obtained from a single cystic fibrosis (CF) patient

Reference number	ID number	Source	API20E	Sample	Resistance profile
[1]	PA-H 1PE (first infection)	Human	2206006	Sputum	Non-MDR
[2]	PA-H 2PCa (mucoid phenotype)	Human	2216004	Sputum	Non-MDR
[3]	PA-H 3BFa (chronic infection)	Human	2206046	Sputum	XDR
[4]	PA-H 3BFb (chronic infection)	Human	2217046	Sputum	XDR
[5]	PA-H 3BFc (chronic infection)	Human	2206046	Sputum	XDR

**Table 3:** *Pseudomonas aeruginosa* animal clinical isolates

Reference number	ID number	Source	API20E	Sample	Resistance profile
[1]	PA-VET 7	Dog	2206006	Auricular swab	MDR
[2]	PA-VET 9	Dog	2202001	Auricular swab	MDR
[3]	PA-VET 10	Dog	2216046	Auricular swab	MDR
[4]	PA-VET 11	Dog	2206046	Auricular swab	MDR
[5]	PA-VET 13	Dog	2206046	Liver	MDR
[6]	PA-VET 15A	Dog	2206006	Auricular swab	MDR
[7]	PA-VET 15B	Dog	2212004	Auricular swab	MDR
[8]	PA-VET 16	Dog	2206046	Auricular swab	MDR
[9]	PA-VET 17	Dog	2206046	Urine	MDR
[10]	PA-VET 18	Dog	2206046	Auricular swab	MDR
[11]	PA-VET 19	Dog	2206046	Auricular swab	MDR
[12]	PA-VET 20A	Dog	2206006	Urine	MDR
[13]	PA-VET 20B	Dog	2216004	Auricular swab	MDR
[14]	PA-VET 22	Dog	2206046	Auricular swab	MDR
[15]	PA-VET 23	Dog	2206006	Auricular swab	MDR
[16]	PA-VET 24	Dog	2206046	Foreskin swab	MDR
[17]	PA-VET 26	Dog	2216046	Auricular swab	MDR
[18]	PA-VET 27	Dog	2217046	Auricular swab	MDR
[19]	PA-VET 28	Dog	2206046	Auricular swab	MDR
[20]	PA-VET 29	Dog	2206046	Auricular swab	MDR
[21]	PA-VET 30	Dog	2206006	Auricular swab	MDR
[22]	PA-VET 31	Dog	2206046	Auricular swab	MDR
[23]	PA-VET 32	Dog	2206006	Auricular swab	MDR
[24]	PA-VET 33	Dog	2212046	Auricular swab	MDR
[25]	PA-VET 34	Dog	2206006	Auricular swab	MDR
[26]	PA-VET 35A	Dog	2212046	Foreskin swab	MDR
[27]	PA-VET 35B	Dog	2210004	Auricular swab	MDR
[28]	PA-VET 36	Dog	2206006	Auricular swab	MDR
[29]	PA-VET 37	Dog	2206006	Auricular swab	MDR
[30]	PA-VET 38	Dog	2210004	Auricular swab	MDR

### Peptide

The peptide AMP2041 used in this study was developed as described elsewhere [9], and synthesized from SelleckChem (Houston, TX, USA). The purity (>98%), sequence and concentration of the peptide were determined and verified by SelleckChem by using high pressure liquid chromatography (HPLC) and mass spectroscopy. The peptide was dissolved in phosphate buffer (PB) (10 mM, 0.8709 g/l K_2_HPO_4_, 0.6804 g/l KH_2_PO_4_, pH 7.0) at the concentration of 1 mg/ml.

### Antibacterial activity evaluation

Methods were described in detail elsewhere [9]. Briefly, bacterial suspension was prepared in PB 10 mM measuring spectrophotometrically the absorbance at 600 nm to a concentration of 10^8^ colony-forming units (CFU)/ml. The adjusted bacterial suspension was then diluted to obtain a final concentration of bacteria of approximately 5 × 10^5^ CFU/ml. Serial dilutions of peptide were performed in a microtiter plate so that final concentrations were within the range 0.4–100 μg/ml. After bacterial suspension addition, microtiter plates were incubated for 2 h at 37 °C. Then, 20 μl of each dilution were plated onto tryptose agar containing 5% bovine erythrocytes. After 24 h of incubation at 37 °C, the colonies were counted. The minimal bactericidal concentration (MBC) was the lowest concentration of peptide that killed >99.9% of bacteria, while lethal dose 90% (LD_90_) was the concentration of peptide that killed 90% of bacteria.

### Time-kill assay

To evaluate the bactericidal kinetic, 5 × 10^5^ CFU/ml of *P. aeruginosa* were incubated with 12.5 μg/ml of AMP2041 at 37 °C in PB. The concentration of 12.5 µg/ml was chosen because it represents the minimal concentration of peptide capable to kill all the tested bacterial strains. Moreover, the same concentration was used for the time-kill assay performed in a previous work on *P. aeruginosa* ATCC 27853 [9]. Aliquots of 20 μl were withdrawn at different intervals (every 5 min until 30 min, then every 10 min until 60 min, then every 30 min until 120 min) and plated onto tryptose agar containing 5% bovine erythrocytes. After overnight incubation at 37 °C, the CFU were counted. Controls were performed in PB without peptide.

### Permeation of the bacterial inner membrane

To assess the ability of antimicrobial peptides to alter the permeability of the inner membrane (IM) of *P. aeruginosa*, a dead-cell stain procedure, using the cationic DNA-staining dye propidium iodide (PI) (Invitrogen, Carlsbad, CA, USA), was performed. PI is unable to permeate the membranes and therefore does not enter viable cells with intact membranes. In dead cells PI gain access to nucleic acids, intercalates between the bases and red fluorescence increases. The stain-dead assay was performed as described in a previous work [11] using 10^9^ CFU/ml log-phase cultures of *P. aeruginosa* ATCC 27853, PA-H 24 and PA-VET 38 in the presence of 12.5 μg/ml of AMP2041 and of 3 μM PI. After peptide addition, the fluorescence emission of PI was measured every 5 min up to 25 min, by a fluorescence microscope (Nikon Eclipse 50i) at 1000×. 4′,6-diamidino-2-phenylindole (DAPI) (Invitrogen, Carlsbad, CA, USA) was used for counterstaining (blue fluorescence). Negative and positive controls (not shown) were obtained in absence of peptide and in presence of 1 mM ethylenediaminetetraacetic acid and 0.5% Triton X-100, respectively.

### Scanning electron microscopy analysis

The test was performed on the *P. aeruginosa* ATCC 27853 reference strain. The cell/peptide ratio used for the scanning electron microscopy (SEM) assay was at least 20 times higher than in the conditions used to determine the MBC. After a contact time of 30 min with AMP2041, the bacterial pellet was obtained by centrifugation at 4000*g* for 5 min and washed twice in PB, pH 7.2. Bacteria were then fixed in a solution of 1% glutaraldehyde in 0.1 M sodium cacodylate (Santa Cruz Biotech, Santa Cruz, CA) for 1 h and washed with water for 1 h. Then, the bacteria were soaked again in water, and the pellet after centrifugation was dehydrated in a series of ethanol washes. Ten microliters of the bacterial suspension were then mounted and imaged.

## Results

### Antibacterial activity evaluation

**Fig. 1:**
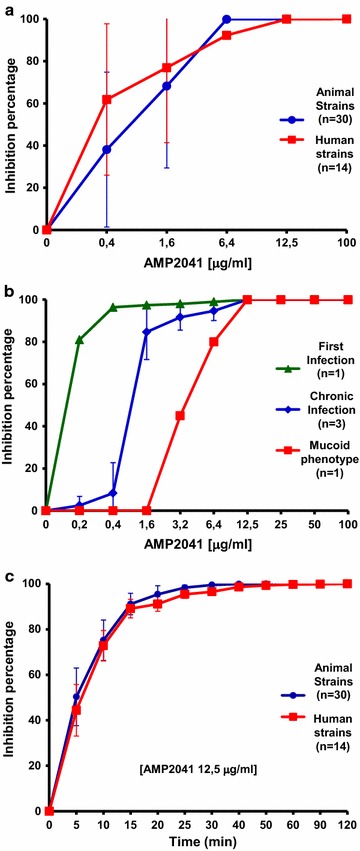
Antimicrobial activity of AMP2041 on **a** *P. aeruginosa* clinical isolates and **b** *P. aeruginosa* isolates from a patient with cystic fibrosis. **c** Time-kill assay

The antibacterial activity of AMP2041 on the 49 clinical isolates of *P. aeruginosa* is shown in Fig. 1a, b. AMP2041 showed antibacterial activity against the tested clinical strains with an LD_90_ ranging from 1.69 to 3.3 μg/ml for animal and human strains, respectively (Table 4). The LD_90_ confidence interval 95% estimated for animal isolates was more narrow (1.14–2.25 μg/ml) compared to human strains (1.75–4.31 μg/ml). The activity of AMP2041 against *P. aeruginosa* strains derived from a single patient affected by CF is reported in Table 4 and Fig. 1b.

**Table 4:** Antimicrobial activity of AMP2041 against PA isolates

Reference number	Human clinical isolates LD_90_ [μg/ml]	Cystic fibrosis isolates LD_90_ [μg/ml]	Animal clinical isolates LD_90_ [μg/ml]
[1]	3.02	0.3	1.09
[2]	1.03	10.4	8.30
[3]	0.6	4.5	0.77
[4]	1.14	3.8	0.93
[5]	0.18	3.2	2.91
[6]	6.07	–	2.87
[7]	7.78	–	2.94
[8]	3.77	–	1.75
[9]	3.63	–	1.75
[10]	0.97	–	0.94
[11]	2.14	–	2.80
[12]	6.15	–	0.99
[13]	0.95	–	2.33
[14]	5.04	–	1.41
[15]	–	–	1.06
[16]	–	–	1.16
[17]	–	–	1.01
[18]	–	–	0.56
[19]	–	–	0.77
[20]	–	–	0.63
[21]	–	–	1.39
[22]	–	–	0.98
[23]	–	–	1.14
[24]	–	–	0.36
[25]	–	–	2.15
[26]	–	–	0.53
[27]	–	–	2.87
[28]	–	–	2.80
[29]	–	–	0.51
[30]	–	–	0.83
Mean ± SD [95% CI]	3.3 ± 2.44 [1.75–4.31]	4.44 ± 3.31 [1.54–7.34]	1.69 ± 1.5 [1.14–2.25]

*LD*_*90*_ lethal dose 90%, *SD* standard deviation, *95% CI* 95% confidence interval

For first infection strain (PA-H 1PE) we observed a value of LD_90_ less than 1 μg/ml. For chronic infection strains (PA-H 3BFa, PA-H 3BFb and PA-H 3BFc) and mucoid phenotype strain (PA-H 2PCa), we observed a shift of LD_90_ towards higher values, ranging from 2.25 to 10.4 μg/ml. In particular, the highest LD_90_ was observed for the mucoid phenotype (10.4 μg/ml). For chronic infection strains as well as for the mucoid phenotype strain, AMP2041 was able to kill all the bacteria at a concentration of 12.5 μg/ml (Fig. 1b).

### Time-kill assay

Inhibition percentages of AMP2041 over time on the 14 *P. aeruginosa* clinical human isolates and on the 30 *P. aeruginosa* clinical animal isolates are shown in Fig. 1c. A reduction of CFU count >90% was observed within 20 min of incubation with peptide.

### Permeation of the bacterial inner membrane

**Fig. 2:**
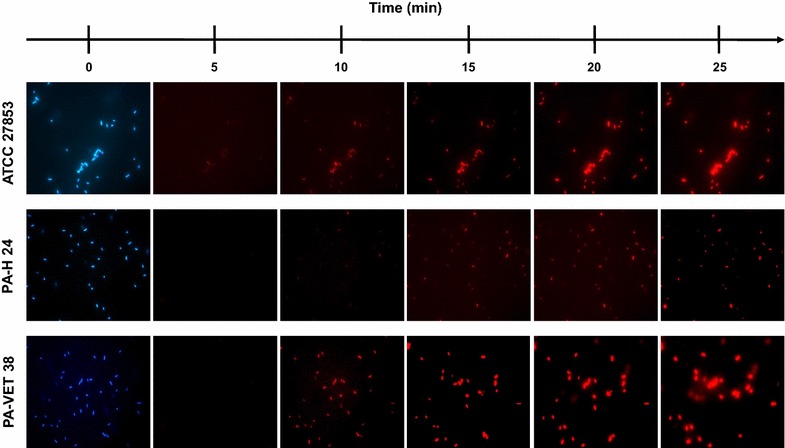
Propidium iodide dead-cell stain assay: permeabilization of the inner membrane of *P. aeruginosa* strains following contact with AMP2041

The stain-dead assay showed a clear red fluorescence after 10 min for *P. aeruginosa* ATCC 27853 and for the animal isolate PA-VET 38, whilst the presence of fluorescence for the human isolate PA-H 24 was not clearly evident before 15 min of incubation (Fig. 2).

### Scanning electron microscopy analysis

**Fig. 3:**
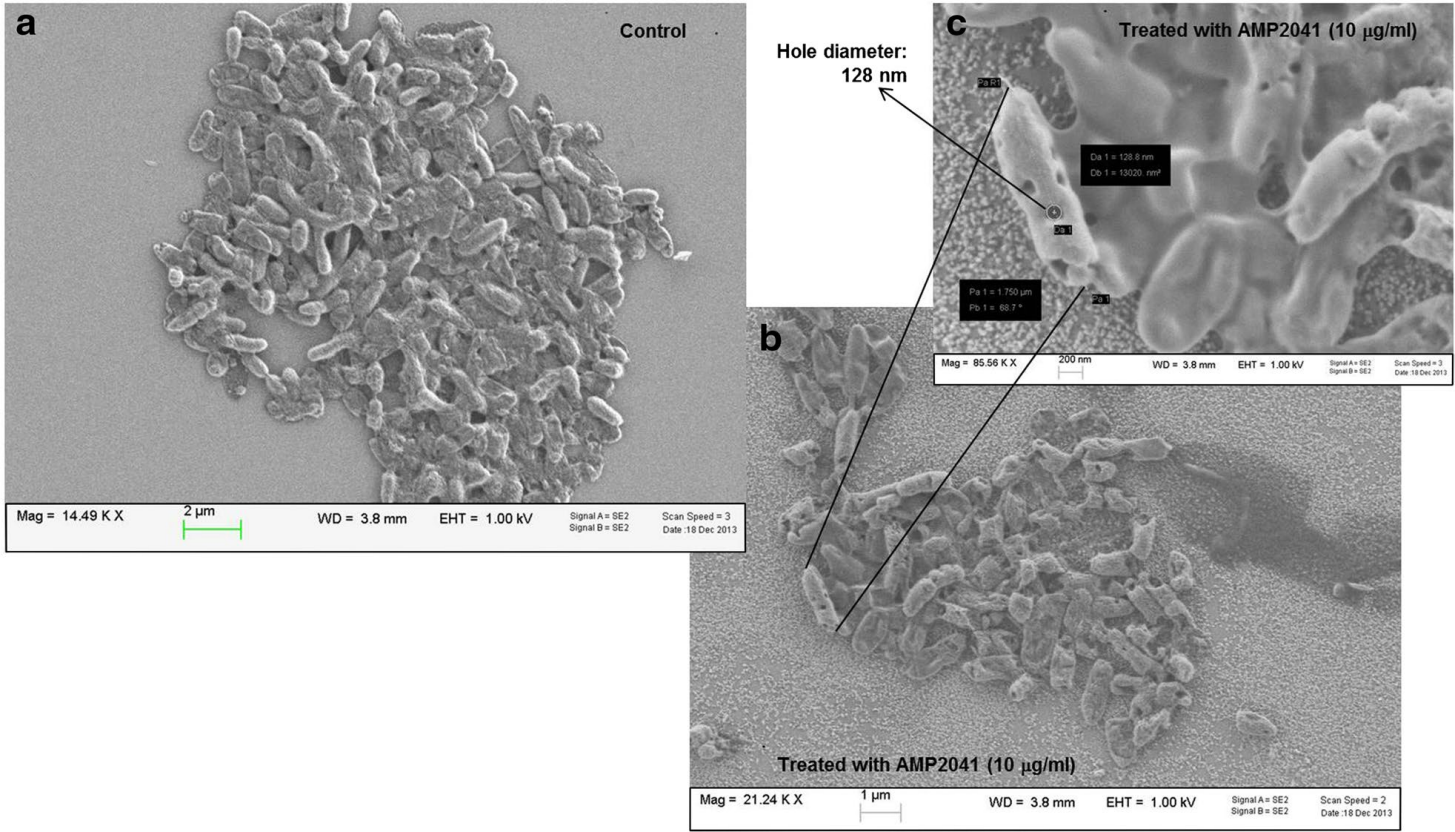
SEM analysis performed on *P. aeruginosa* ATCC 27853. **a** Untreated; **b** treated with AMP2041; **c** holes size measurement

The SEM analysis was performed on the *P. aeruginosa* ATCC 27853 reference strain. The untreated bacteria displayed a smooth and intact surface (Fig. 3a) with typical rod morphology about 2 µm long and 0.5 µm wide. After incubation with AMP2041, bacteria showed several holes, multiple dents and bursts with deep craters throughout cell wall (Fig. 3b, c). Lysed cells and debris were also observed.

## Discussion

*Pseudomonas aeruginosa* is an ubiquitous organism. Its ability to survive on minimal nutritional requirements and to tolerate a variety of physical conditions allows its persistence in both community and hospital settings [12]. *P. aeruginosa* is a serious therapeutic challenge for treatment of both community-acquired and nosocomial infections, due to the ability of this microorganism to develop resistance to multiple classes of antibacterial agents, even during the course of therapy [13, 14]. The increasing frequency of MDR or XDR *P. aeruginosa* strains is of concern as effective antimicrobial options are limited [15, 16]. Moreover, only a few new antibiotics are currently under development [6]. An increase in MDR bacterial infections among companion animals has been documented in multiple veterinary hospital settings [17]. This is of particular importance due to the risk of transmission to humans and other companion animals in close contact with infected animals, even because in our countries the pet population continues to rise and the contacts between people and their companion animals grows stronger [18–20]. Therefore, the discover of new agents or innovative approaches able to counteract the growing problem of antimicrobial resistance become crucial.

The synthetic peptide AMP2041 is a cationic peptide and possesses a significant proportion of hydrophobic or non-polar residues. These structural features are common to many antimicrobial peptides [9, 21]. The hydrophobic core is essential for the antimicrobial peptide to effectively permeate the bacterial membrane. The hydrophobic core is flanked at both ends by cationic and polar residues that help to solubilize the peptides in aqueous solution. Cationic and polar residues are also important for the initial electrostatic attraction of antimicrobial peptides to negatively charged phospholipid membranes of bacteria. Also the conformation assumed by AMP2041 might be responsible for the observed antimicrobial activity [8, 9, 22].

Antimicrobial activity of AMP2041 on human clinical isolates was never investigated before. In this study we evaluated the activity of AMP2041 on 19 MDR or XDR *P. aeruginosa* strains isolated from different pathological conditions of humans, among which five deriving from a CF patient. Moreover, the activity of AMP2041 was tested on a sample of 30 MDR *P. aeruginosa* strains derived from dog otitis. AMP2041 showed an excellent activity against all the examined strains. In particular, on average, it was more effective against animals strains, with an LD_90_ of 1.69 μg/ml and an MBC of 6.4 μg/ml (3.2 μM), compared with human strains, LD_90_ of 3.3 μg/ml, MBC of 12.5 μg/ml (6.2 μM). The antimicrobial activity found here for AMP2041 against *P. aeruginosa* is comparable or better than many highly-active antimicrobial peptides. Zhou et al. have found MIC values against *P. aeruginosa* ranging from 31 to >256 μg/ml for peptides synthesized via ring-opening polymerization of α-amino acid *N*-carboxyanhydrides [23]. Regarding the activity of Cecropin A, an insect antimicrobial peptide, against *P. aeruginosa*, a MIC value of 64 μg/ml is reported by Zhou et al. [23] and a lethal concentration of 3.5 μM by Andreu et al. [24]. For PR-39, an antimicrobial peptide from pig intestine, a lethal concentration of 200 μM against *P. aeruginosa* was reported [25]. Very recently, minimum lethal concentrations ranging from 3 to 100 μM were reported for *E. coli* MreB derived antimicrobial peptides against *P. aeruginosa* [26].

It is noteworthy the antimicrobial activity of AMP2041 against the strains derived from the patient with CF. Most patients with CF become chronically infected with wild-type (first infection) *P. aeruginosa* strains early in their life. During the years following the initial colonization, the first infection strains may mutate into mucoid variants [27, 28]. Conversion to the mucoid phenotype is thought to be driven mainly by the unique CF microenvironment [28]. In our case, the *P. aeruginosa* mucoid strain was less sensitive to AMP2041 than the other tested CF strains (Fig. 1b). However, this result was obtained on a single strain and should be further investigated with a wider sample to confirm a higher resistance of the mucoid phenotype compared to the first and chronic infection isolates. The observed lower sensitivity to AMP2041 of the *P. aeruginosa* mucoid strain could be linked to the over production of mucoid exopolysaccharides that hide the negatively charged surface components to which positively charged peptides are attracted.

Mean MBC for dog strains (n = 30, MBC = 6.4 μg/ml—see Fig. 1a) is higher than values previously found for the reference strain ATCC 27853 (4.35 μg/ml) and other dog isolates (n = 6, MBC = 2.44 μg/ml) [8]. Therefore, the increased sample size allowed us to re-evaluate the MBC average value previously obtained.

The bacterial killing assay indicated a CFU reduction >90% within 20 min (Fig. 1c). Therefore, the antimicrobial activity of AMP2041 occurs quickly and the killing kinetic profiles of human and animal clinical isolates, never investigated before, were similar to that previously reported for *P. aeruginosa* ATCC 27853 [8, 9]. These results were almost unrelated to the sources of strains, suggesting that the mechanism of action was similar for all the examined strains. The killing kinetics are comparable [29] or better [30] than those obtained with other established antimicrobial peptides at their lethal concentration. Saikia et al., instead, showed that two out of four *E. coli* MreB derived peptides completely killed *P. aeruginosa* within 5 min of treatment with the peptides at their minimum lethal concentrations [26]. However, in our case the kinetic of killing of AMP2041 was derived from the testing of many different *P. aeruginosa* strains, while in the other cases only one *P. aeruginosa* strain was tested. Moreover, an interesting fact that emerges from the work of Saikia et al. is that for *P. aeruginosa* there is no direct correlation between the minimum lethal concentration and the rapidity of killing, because the peptide with the best minimum lethal concentration (3 μM) completely killed the bacteria only after 120 min.

The stain-dead assay was performed on the *P. aeruginosa* ATCC 27853 reference strain and on PA-H 24 and PA-VET 38, which were selected for the assay as representative of strains with a high level of antibiotic resistance, being XDR and MDR (see Tables 1, 3), respectively. Results indicated that the inhibitory effect of AMP2041 is linked to an altered permeability of the cellular membrane of *P. aeruginosa* (Fig. 2). This is in accordance with the mechanism of action of cationic antimicrobial peptides which cause cell death through loss of membrane integrity [22]. The timing of the occurrence of red fluorescence was in accordance with time kill results. The membrane damage was evident within 10 min of incubation for the reference strain and the animal isolate PA-VET 38 and within 15 min for the human isolate PA-H 24.

To confirm that the fluorescence increase was due to morphological changes of bacterial membrane, a SEM analysis was performed on the *P. aeruginosa* ATCC 27853 reference strain treated with AMP2041. SEM analysis provided evidence for a direct membrane damage, showing the presence of several holes, dents and bursts throughout cell wall (Fig. 3b, c). Similar membrane changes are also described for other cationic antimicrobial peptides [23, 26, 31]. The microbicidal effect of AMP2041 was also confirmed by the presence of lysed cells.

## Conclusions

In conclusion, we assessed the good antimicrobial activity of AMP2041 on *P. aeruginosa* strains of human origin, including those deriving from a CF patient. Moreover, we confirmed the excellent antimicrobial activity of AMP2041 on *P. aeruginosa* strains derived from dog otitis. We also assessed that AMP2041 antimicrobial activity is linked to changes of the *P. aeruginosa* cell wall morphology and the increasing of membrane permeability. This mechanism of action is less prone to induce resistance by the pathogen compared to antimicrobials acting against intracellular targets. However, clinical trials with adequate animal models should be performed to define the therapeutic potential of AMP2041.

## Abbreviations

CF: cystic fibrosis
CFU: colony-forming unit
DAPI: 4′,6-diamidino-2-phenylindole
ECDC: European Centre for Disease Prevention and Control
EDTA: ethylenediaminetetraacetic acid
ESKAPE: *Enterococcus faecium* (E) *Staphylococcus aureus* (S) *Klebsiella pneumoniae* (K) *Acinetobacter baumannii* (A) *Pseudomonas aeruginosa* (P) *Enterobacter* Species (E)
HPLC: high pressure liquid chromatography
IM: inner membrane
LD: lethal dose
MBC: minimal bactericidal concentration
MDR: multi-drug resistant
PA-H: *Pseudomonas aeruginosa*, human isolate
PA-VET: *Pseudomonas aeruginosa*, animal isolate
PB: phosphate buffer
PDR: pan-drug resistant
PI: propidium iodide
SEM: scanning electron microscopy
XDR: extensively-drug resistant
